# Impact of mobile phone usage on empowerment of rural women entrepreneurs: Evidence from rural Bangladesh

**DOI:** 10.1016/j.heliyon.2023.e21604

**Published:** 2023-10-31

**Authors:** Md Sadekur Rahman, Md Enamul Haque, Md Safiul Islam Afrad, Shaikh Shamim Hasan, Md Abiar Rahman

**Affiliations:** a Department of Agricultural Extension, Hajee Mohammad Danesh Science and Technology University (HSTU), Dinajpur 5200, Bangladesh; b Department of Agricultural Extension and Rural Development, Bangabandhu Sheikh Mujibur Rahman Agricultural University (BSMRAU), Gazipur 1706, Bangladesh; c Department of Agroforestry and Environment, Bangabandhu Sheikh Mujibur Rahman Agricultural University (BSMRAU), CIFOR-ICRAF Bangladesh Office, Gazipur 1706, Bangladesh

**Keywords:** ICT, Rural entrepreneur, Women empowerment, Farm women, Empowerment dimensions

## Abstract

Mobile phones have emerged as the predominant technology in developing countries, especially within agricultural enterprises. This research investigates the influence of mobile phone utilization on the empowerment of female entrepreneurs in rural farming communities. Data were gathered from 150 female agricultural entrepreneurs in the rural regions of Bangladesh. The empowerment of these women was assessed across five dimensions: economic, social, technological, psychological, and political. The findings indicate that the composite empowerment index was notably higher at 61.92% post-mobile phone adoption, in contrast to 37.47% prior to its use. This underscores the positive impact of mobile phone utilization on the empowerment of rural female farmers. The research highlighted significant enhancements in all five empowerment dimensions, with these augmentations being statistically meaningful. The most pronounced improvement was observed in economic empowerment, while political empowerment witnessed the least growth. Furthermore, the shifts were profoundly correlated with the other three dimensions: social, technological, and psychological. The evidence establishes a link between mobile phone adoption by rural female farmers and their subsequent empowerment, with empowerment metrics showing a steady rise from the low-usage to the high-usage categories. Consequently, the correlation is positive. The results advocate that mobile phone utilization assists rural female farmers in augmenting their empowerment. It is thus recommended that policymakers and developmental organizations prioritize mobile technology as a tool to empower rural women in Bangladesh. This can be realized by enhancing accessibility, confronting socio-cultural barriers, and fostering digital literacy.

## 1 Introduction

The potential of mobile technology to expedite social and economic development is increasingly recognized, particularly when directed towards the empowerment of women. Mobile phones, as a conduit for economic growth, can significantly enhance the well-being of families and communities when women are the primary beneficiaries [1]. Despite the ubiquity of mobile technology across diverse populations, its nexus with women's empowerment in developing nations has become pivotal in shaping global development strategies. The Sustainable Development Goal (SDG) 5 emphasizes the need “to achieve gender equality and empower all girls”, with sub-target 5b advocating for the “enhancement of enabling technology, especially information and communications technology, to bolster women's empowerment”. This emphasis stems from the recognized interplay between technology and development, which has profound implications for the theory and praxis of women's empowerment [2]. Contemporary research underscores the tangible benefits women accrue from mobile usage, ranging from an enhanced sense of societal relevance to challenging and redefining traditional gender norms, thereby fostering improved psychological well-being [3].

However, empowerment is often predominantly conceptualized from a socioeconomic lens. Discussions surrounding gender (in)equity have highlighted disparities in women's access to and use of mobile phones within the technological realm. Paradoxically, mobile phones have been associated with both empowerment and disempowerment outcomes, leading to an uneven distribution of resources along gender lines and exacerbating existing gender disparities [4]. In patriarchal settings, women's access to mobile phones is often curtailed [5]. Domestic responsibilities may restrict mobile phone usage among dependent women, while migrant workers leverage mobile communication to navigate maternal identities amidst pressures from families left behind [6].

Empowerment, at its core, is the process of augmenting an individual's or a group's capacity to make informed decisions and actualize those decisions into tangible actions and outcomes. Central to this process are actions that augment both individual and community resources while enhancing the efficacy and equity of the overarching organizational and institutional frameworks governing these resources [7]. Mobile technology can serve as a catalyst for women's empowerment by broadening their knowledge base, fortifying their social networks, and diversifying their socioeconomic opportunities [8]. Empowered women exert greater control over tangible and intangible resources, ranging from financial assets to knowledge, information, and decision-making authority at various societal levels [9]. Information empowers rural female farmers, enabling them to actively participate in decision-making and share insights with peers [10].

In Bangladesh, women have historically played a pivotal role in agricultural endeavors. Predominantly, rural women are deeply entrenched in labor-intensive agricultural tasks, spanning from sowing to harvesting and encompassing myriad post-harvest activities [11]. Effective utilization of mobile technology can empower women to adeptly manage agricultural resources, optimize farm enterprises for sustenance, and seamlessly access markets for trade [12]. While mobile phones offer farmers the autonomy to directly sell their produce, bypassing intermediaries who often command a disproportionate share of profits, women's access to this technology remains limited. A significant number of women lack even rudimentary access to ICTs, thereby missing out on these opportunities [6]. Nonetheless, numerous reports underscore the empowering potential of mobile technology for women, facilitating financial self-sufficiency, mutual support across various sectors, and access to real-time market information [13]. Mobile technology has revolutionized the way individuals engage with the world, enabling farmers to access timely information on weather patterns, agricultural methodologies, pest control, market trends, and more, thereby optimizing agricultural outcomes [14,15].

Mobile phones have facilitated women's integration into globalized, modernized, and neoliberal economic structures, as evidenced by the proliferation of garment factories, microcredit initiatives, labor migration, and contemporary agricultural practices [16]. Despite the meteoric rise in mobile communication technology, evidence suggests that its integration into farming activities (e.g., agriculture, fishing) has enhanced professional communication within communities [17]. Collaborative efforts between governmental and non-governmental organizations (NGOs) have empowered rural women to spearhead micro and small-scale enterprises, encompassing dairy and poultry farming, handicrafts, daily vending, and more [18]. Micro-enterprises, typically unregistered and informally operated, often cater to economically disadvantaged populations [19]. These enterprises may also adopt a familial model, with kin playing integral roles in their operations [20]. The development of such enterprises presents a promising avenue for sustainable livelihoods among rural households.

In rural Bangladesh, women commonly engage in entrepreneurial activities such as poultry and livestock rearing, traditional handicrafts, small-scale retail, village phone operations, vegetable and fruit cultivation and distribution, traditional sweet dish production, rice/paddy trading, rickshaw leasing, clothing ventures, among others [21]. Poultry, dairy, nursery, rice husking, fisheries, and handicraft sectors are integral to Bangladesh's agrarian and subsistence economy, providing essential nutrition and generating income and employment opportunities [22]. Poultry farming, for instance, not only augments the income of the farming community but also offers employment avenues for the unemployed and widows in rural locales, fostering self-reliance [23]. It is universally recognized that sustainable development is unattainable without the active participation of women. The involvement of rural women in diverse small-scale enterprises is anticipated to have a transformative impact on their lives across personal, social, and economic spheres by amplifying their access to and control over resources [24]. Furthermore, women's contributions to household income are poised to bolster their role in household decision-making, thereby significantly empowering them. Consequently, there exists a symbiotic relationship between entrepreneurial development and empowerment.

For micro-entrepreneurs, communication is paramount when liaising with suppliers, customers, traders, and occasionally, intermediaries. Activities such as price negotiations, payment term discussions, inventory management, and delivery scheduling necessitate precise and timely communication. Often, micro-entrepreneurs resort to intermediaries to facilitate the sale of their agricultural produce or handicrafts. A quintessential example in Bangladesh involves rice traders who, in the absence of direct information, depend on intermediaries to ascertain current rice prices or varieties [25]. In such contexts, mobile phones have emerged as indispensable tools, empowering rural women to establish micro-enterprises. These devices not only facilitate business operations but also catalyze productivity, networking, and information dissemination. The ability to execute transactions or place orders via mobile phones obviates the need for physical interactions, thereby bridging temporal and spatial divides. For many micro-entrepreneurs, mobile phones function as conduits for information collection and dissemination, especially for stakeholders like fishermen or female farmers seeking price points, supplier details, or potential customers [26]. Additionally, mobile phones foster networking opportunities, where serendipitous interactions can potentially culminate in novel business ventures.

While numerous studies have elucidated the influence of ICT on entrepreneurship [21,27,28], empowerment [7,17,29], mobile phone utilization for entrepreneurship [30,31], and women's empowerment [20,23,32], there remains a lacuna in understanding the mechanisms through which mobile phone usage augments the empowerment of female entrepreneurs. Given the pivotal role of female entrepreneurial empowerment, especially in rural Bangladesh, there is an exigent need to delve into this domain to inform policy decisions. Therefore, this study explores the impact of mobile phone usage on rural farm women entrepreneurs' empowerment.

This article proffers several salient contributions to the extant academic discourse on the nexus between mobile technology and women's empowerment in developing regions. Primarily, it furnishes empirical evidence delineating the direct correlation between mobile phone adoption and the empowerment of rural female entrepreneurs, assessing empowerment across multifaceted dimensions such as economic, social, technological, psychological, and political. Furthermore, by focusing on rural Bangladesh—a region where women, despite their extensive involvement in labor-intensive agricultural activities, grapple with marginalization and resource constraints—this study provides nuanced insights pivotal for crafting targeted interventions and policies. The research endeavors to bridge the existing scholarly void by dissecting the intricate dynamics through which mobile phone usage can bolster female entrepreneurial empowerment. Emphasis is placed on the pragmatic ramifications of mobile phone adoption in challenging and reconfiguring entrenched gender norms, and in countering the impediments posed by patriarchal systems and gender imbalances. Consequently, this study stands as an invaluable repository for academics, policymakers, and practitioners dedicated to championing gender parity and women's empowerment in low-income nations.

## 2 Methodology

### 2.1 Locale of the study

**Fig. 1:**
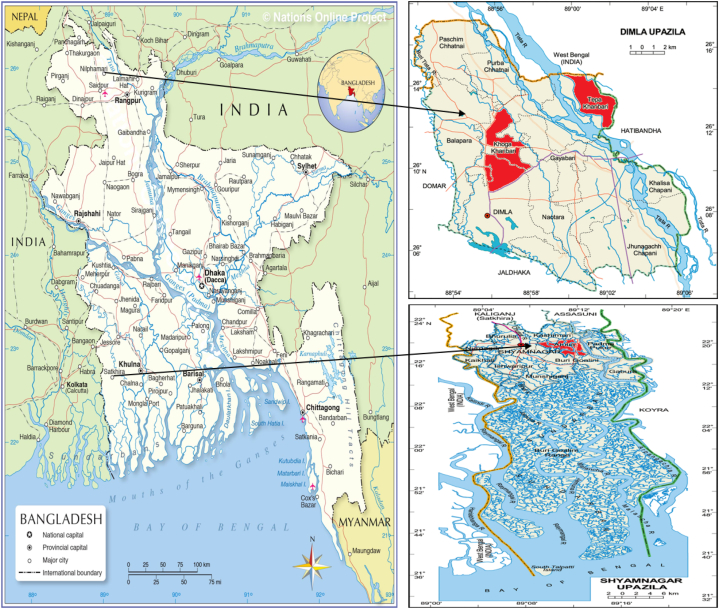
The study areas in Bangladesh.

This research was undertaken in two geographically distinct regions: Dimla (26.1278°N 88.9250°E) situated in Nilphamari and Shyamnagar (22.3306°N 89.1028°E) located in the Satkhira district. These locales were purposefully chosen due to the observable uptick in mobile phone adoption among rural farm women. The surge in mobile phone ownership and associated services, especially catalyzed by interventions from non-governmental organizations (NGOs), is noteworthy. In Nilphamari, the predominant populace consists of agricultural laborers, day laborers, farmers, and small-scale entrepreneurs, with a significant inclination towards cattle rearing. The primary economic undertakings in both districts are anchored in agriculture, encompassing crop cultivation, livestock husbandry, and aquaculture. Since 2015, mobile phone-centric interventions have been operationalized in these regions by two local NGOs: Pollisree in the northern segment and Sushilon in the southern, in collaboration with Oxfam Bangladesh. Pollisree has instituted Community Based Organizations (CBOs) primarily comprising rural farm women engaged in crop-centric endeavors in Tepa Kharibari and Khoga Kharibari unions. These women also address issues pertinent to livestock farming. Analogously, Sushilon has established CBOs actively participating in fisheries and related ventures. In the northern expanse of Bangladesh, Dakkhin Charivari village of Tepa Kharibari union and Doholpara village of Khoga Kharibari union within Dimla Upazila of Nilphamari district were designated as the research locales. In contrast, in the southern region, Borokupot village of Atulia union within Shyamnagar Upazila of Satkhira district was selected, focusing on rural women engaged in fisheries enterprises. For enhanced clarity and geographical comprehension, Fig. 1 provides maps delineating the specific study locations.

### 2.2 Data sources and collection

Within the context of this study, rural farm women, who employ mobile phones across diverse phases of their agricultural ventures—from inception to harvest, post-harvest processes, and marketing—are conceptualized as individual farm entrepreneurs. An initial comprehensive list of the target population was curated in collaboration with pertinent local administrative personnel. Subsequently, samples were ascertained employing the field technique delineated by Thakur and Chander's [30]. The research encompassed rural farm women entrepreneurs engaged in crop, livestock, and fisheries sectors, leveraging mobile phones for communication and farm-related information dissemination. Utilizing a disproportionate stratified sampling methodology, a cohort of 150 rural farm women was earmarked for the study.

For the acquisition of quantitative data, in-person interviews were orchestrated utilizing a meticulously structured and pre-validated questionnaire. Qualitative data were amassed employing Rapid Rural Appraisal instruments, including Focus Group Discussions, Case Studies, and Participatory Rural Appraisal tools across both research locales. To ensure clarity and foster rapport, the researcher, in conjunction with Upazila Agriculture Officers, Agricultural Extension Officers, and NGO representatives, meticulously refined topics and queries. Data collection transpired from August to December 2020.

### 2.3 Measurement of empowerment of rural farm women

Empowerment was operationalized using five salient indicators: economic, social, technological, psychological, and political, drawing inspiration from extant literature [17,[33], [34], [35]]. Each indicator was gauged using a 4-point rating scale, and corresponding indices were formulated to encapsulate the degree of empowerment within each dimension. T-tests were employed to discern differences in the mean values of empowerment pre- and post-mobile phone adoption.

#### 2.3.1 Measurement of economic empowerment

Economic empowerment is delineated by the quantum of income and profit accrued by female entrepreneurs. This dimension encapsulates the access and contributions of rural female entrepreneurs to diverse economic facets, gauged pre- and post-mobile phone adoption. A 4-point rating scale was employed, with scores delineated as: 0 for none, 1 for low, 2 for medium, and 3 for high. The Access to Economic Activities Index (AEAI) was formulated to encapsulate the degree of empowerment within this dimension, drawing methodologies from Sarker [36] and Hossain [37]. A *t*-test was conducted to differentiate changes in the mean value of economic empowerment pre- and post-mobile phone adoption.

#### 2.3.2 Measurement of social empowerment

Social empowerment is characterized by the level of esteem and acknowledgment a female entrepreneur garners within her community. This dimension encompasses the involvement of rural women in a myriad of social issues, assessed pre- and post-mobile phone adoption using a 4-point rating scale. The Participation in Social Activities Index (PSAI) was formulated to encapsulate the degree of empowerment within this dimension, drawing methodologies from Sarker [36], Hossain [37], and Khan et al. [38]. A *t*-test was employed to discern differences in the mean value of social empowerment pre- and post-mobile phone adoption.

#### 2.3.3 Measurement of technological empowerment

Technological empowerment pertains to access to information, knowledge, competencies, and resources. This dimension gauges the extent of rural women's access to diverse technological facets pre- and post-mobile phone adoption. The Access to Technological Activities Index (ATAI) was formulated to encapsulate the degree of empowerment within this dimension, drawing methodologies from Sarker [36], Hossain [37], and Khan et al. [38]. A *t*-test was conducted to discern differences in the mean value of technological empowerment pre- and post-mobile phone adoption.

#### 2.3.4 Measurement of psychological empowerment

Psychological empowerment is conceptualized as an individual's sense of self-efficacy, manifesting as self-assured behavior. This dimension gauges shifts in rural women's behaviors across various psychological facets pre- and post-mobile phone adoption. The Psychological Well-being Index (PWI) was formulated to encapsulate the degree of empowerment within this dimension, drawing methodologies from Sarker [36], Hossain [37], and Khan et al. [38]. A *t*-test was used to compare the mean value of psychological empowerment before and after mobile phone use.

#### 2.3.5 Measurement of political empowerment

Political empowerment is defined as access to decision-making processes, especially those determining the future of rural women entrepreneurs, and includes the ability to vote and to speak up and take collective action [38]. It is measured by the extent of participation of rural women in different political issues before and after mobile phone usage. The Participation to Political Activities Index (PPAI) was developed to represent the level of empowerment in this indicator, based on methodologies adopted from Sarker [36], Hossain [37], and Khan et al. [38]. A *t*-test was performed to compare the mean value of political empowerment before and after mobile phone usage.

#### 2.3.6 Extent of empowerment

To measure empowerment, an empowerment index was developed, incorporating all the indicators and issues addressed in this study. According to Biswas and Kabir [39], there exist two methodologies to formulate the empowerment index: one premised on equal weights and the other on unequal weights. This index underwent development over a two-year span. Initially, an empowerment index for a rural female entrepreneur is derived for a singular indicator, encompassing multiple sub-indicators or facets. Subsequently, a composite empowerment index is devised, amalgamating diverse indicators with equal weights. The Empowerment Index for a rural female entrepreneur, predicated on a singular indicator, assigns equal weights across various facets and is articulated in percentage terms.

In the initial phase, the Empowerment Index for a rural female entrepreneur, predicated on a singular indicator encompassing multiple facets, was computed employing the subsequent formula:

(1) $${\text{EI}}_{\text{ij}}=\frac{X1+X2+\ldots \ldots \ldots .+\text{Xn}}{M}\times 100$$

(2) $$=\frac{\begin{array}{c} \sum \text{Xj}\\ j=1 \end{array}}{M}\times 100$$

where, EI_ij_ = Empowerment index of ith women for jth indicator; Xj = Value of individual issues for jth indicator; M = Highest possible score; and N = Quantity of specific issues of an indicator.

In the second stage, the composite empowerment index, consisting of equal-weighted indicators, is calculated as follows.

(3) $${\text{EI}}_{i}=\frac{\text{EIi}1+\text{EIi}2+\ldots \ldots \ldots .+\text{EIiN}}{N}\times 100$$

(4) $$=\frac{\begin{array}{c} \sum \text{EI}ij\\ j=1 \end{array}}{N}$$

where, EI denotes composite empowerment index of ith women; and N is the number of indicators considered in the composite index.

### 2.4 Statistical analysis

To explore differences across variables, research hypotheses were posited, articulating anticipated disparities. Null hypotheses were also delineated, suggesting no significant variance or contribution between the pertinent variables. The specific null hypotheses, derived from the research questions and objectives, are as follows:

H1 There exists no significant disparity in the empowerment of rural women entrepreneurs pre- and post-mobile phone adoption.

H2 There exists no significant correlation between the extent of mobile phone usage and the empowerment of rural women.

For H1, correlation and t-tests were employed to probe the associations among the five dimensions of women's empowerment. For H2, the Chi-square test was utilized to ascertain the relationship between mobile phone adoption by rural farm women and their empowerment. All analyses were conducted using SPSS Version 20.

### 2.5 Ethical approval

This study was conducted in strict adherence to ethical standards and procured approval from the ethical review committee of Bangabandhu Sheikh Mujibur Rahman Agricultural University. Prior to each interview, participants were apprised of the study's objectives and the confidentiality of their responses, and verbal consent was secured.

## 3 Results

### 3.1 Extent of empowerment of rural women entrepreneurs by using mobile phone

#### 3.1.1 Economic empowerment

Economic empowerment is conceptualized as women possessing both the potential for financial growth and the agency to make and act upon financial decisions. The data suggests that prior to mobile phone adoption, women were significantly marginalized in terms of access to economic activities. Specifically, a substantial majority (71%) of the respondents had minimal access, 15% had no access whatsoever, 13% had moderate access, and a mere 1% of the rural women entrepreneurs had extensive access to various economic activities (Table 1). This marginalization was particularly pronounced in terms of their ability to promote agricultural products; 41.0% had no avenue to publicly market their farm products, and 22% lacked the means to order and procure farm inputs without physically visiting markets. An equivalent proportion (21%) of the farm women encountered challenges in sourcing information pertinent to their farms. Similarly, prior to mobile phone adoption, they lacked the facility to securely and swiftly transact money through cashless methods.

**Table 1:** Women entrepreneurs and their access to economic activities.

Sl. #	Economic activities	Level of access to economic activities (%)							
		Before mobile usage				After mobile usage			
		No	Low	Med.	High	No	Low	Med.	High
1.	Access to market (as a buyers and sellers)	3	64	29	4	0	5	52	43
2.	Getting price information of agricultural produce	16	70	14	0	0	19	59	22
3.	Access to financial services and credit facilities	2	85	13	0	0	16	62	22
4.	Allow fast and safe transaction to pay and/or receive money	21	65	12	2	0	20	61	19
5.	Keeps farm enterprise records and not dependent on middlemen or irregular buyers	9	72	15	4	0	19	42	39
6.	Dispersion of agricultural produce prices across the market	9	69	20	2	2	23	52	23
7.	Access to more income-generating activities (IGAs) such as bamboo made product sell, crab culture, and (*Sana*) milk made preparation	11	81	8	0	2	53	37	8
8.	Cost of information search (reduced need for transport/travel)	21	75	4	0	1	57	32	10
9.	Ordering and purchasing farm input	22	74	4	0	11	50	27	12
10.	Promoting agricultural farm products	41	51	8	0	10	55	20	15
	Average	15	71	13	1	3	32	44	21
	Mean of AEAI	33.28				61.42			
	Range of AEAI	16.7–70.0				30.0–93.3			

Scale value: No access = 0, Low access = 1, Medium access = 2, High access = 3.

AEAI = Access to Economic Activities Index.

The overarching dynamics governing rural women entrepreneurs' access to markets and economic activities underwent a significant transformation following the integration of mobile phones. A predominant segment of these women, 44%, secured medium access. Roughly one-third, or 32%, achieved limited access, while 21% realized extensive access to economic activities in rural contexts. In stark contrast to the pre-mobile phone era, a mere 3.0% of women found themselves without any access to economic activities.

**Fig. 2:**
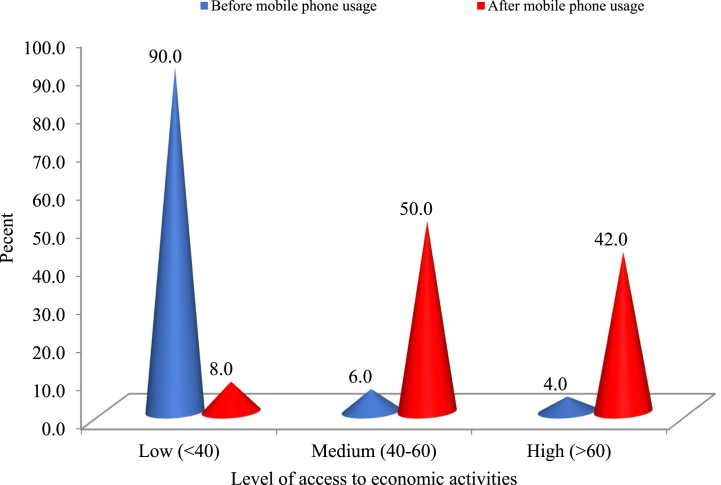
Rural women's access to economic activities index (AEAI).

The vast majority of women were able to penetrate markets and ascertain price data for the agricultural products they cultivated and marketed, thereby amplifying their capacity to harness market intelligence via mobile phones. Instead of being dependent on intermediaries or a restricted cohort of intermittent buyers, rural women harnessed mobile phones to directly negotiate prices with buyers and authenticate rates for their commodities [1]. The Access to Economic Activities Index (AEAI) surged from 16.7% to 70% pre-mobile phone intervention and escalated from 30.0% to 93.3% post-intervention. The AEAI's mean value witnessed a marked elevation from 33.28% pre-mobile phone adoption to 61.42% post-adoption, signifying a pronounced augmentation in the economic empowerment of rural women entrepreneurs [40]. However, a scrutiny of the distribution of rural women entrepreneurs based on their AEAI value reveals palpable disparities across different respondent categories in terms of their economic activity access pre- and post-mobile phone adoption (Fig. 2).

Fig. 2 delineates that, in the era preceding mobile phone adoption, an overwhelming 90.0% of respondents had restricted access to economic activities, 6.0% secured medium access, and a scant 4.0% enjoyed extensive access. Following the integration of mobile phones, access levels witnessed a significant enhancement, with a dominant 92.0% achieving medium to extensive access to economic activities, leaving only 8.0% with limited access. This progression can be attributed to the surmounting of socio-cultural impediments such as entrenched patriarchal norms, coupled with addressing human resource challenges like a dearth of awareness and training on mobile technology and mobility constraints due to geographical confines. Furthermore, governmental endeavors to digitize the nation and champion the merits of mobile technology in rural precincts have catalyzed rural women's expanded access to economic activities [41]. These findings resonate with the insights presented by Nord et al. [42].

#### 3.1.2 Social empowerment

Social empowerment is construed as the acquisition of novel and invaluable knowledge, insights, and cognizance pertaining to a myriad of issues, subjects, and endeavors that resonate with women. This investigation reveals that rural women entrepreneurs manifested subdued engagement in social matters prior to the adoption of mobile phones. In fact, on average, 69% of respondents exhibited limited engagement, 21% showcased moderate engagement, and a trifling 3% displayed extensive engagement in diverse social activities pre-mobile phone adoption (Table 2). Subsequent to the integration of mobile phones, there was a pronounced surge in rural women's engagement in social activities: on average, 73% of rural women exhibited moderate to extensive engagement in social matters, 26% displayed limited engagement, and a mere 2% abstained from social activities altogether. Among the ten social activities surveyed, a majority (64%) of rural women could seamlessly interact with migrant family members or their community, signaling a liberation from familial constraints and culminating in augmented social empowerment.

**Table 2:** Women entrepreneur's participation in social aspects.

Sl. #	Social activities	Level of participation (%)							
		Before mobile usage				After mobile usage			
		No	Low	Med.	High	No	Low	Med.	High
1.	Participation in social functions such as marriage ceremonies, burials, religious activities, *chehlam*, *khatna*etc.	2	57	34	7	0	2	59	39
2.	Participation in social campaign on various issues (vaccination, plantation etc.)	0	53	43	4	0	7	45	49
3.	Helping neighbors and relatives during emergency needs such as delivery, birth, etc.	5	72	15	8	0	4	45	51
4.	Cooperate with neighbors and relatives to provide voluntary help during floods, cattle, fish robbery, fires, etc.	17	49	32	2	0	9	55	37
5.	Maintaining social relation with customer and suppliers	9	68	23	0	5	10	56	29
6.	Communicating and inspiring friends and fellow entrepreneurs	20	65	12	3	0	27	56	17
7.	Participation in group activities with women networks	8	72	20	0	3	20	34	43
8.	Conflict solving capacity/Village *Salish*	3	85	10	2	2	45	44	9
9.	Complaining against social problems such as eve-teasing, dowry, physical assault etc.	2	84	14	0	3	47	43	8
10.	Interacting with family and community migrant worker	9	79	10	2	7	17	12	64
	Average	8	69	21	3	2	26	45	28
	Mean of PSAI	39.71				66.00			
	Range of PSAI	20.0–73.3				30.0–90.0			

Scale Value: No participation = 0, Low participation = 1, Medium participation = 2 High participation = 3; PSAI= Participation to Social Activities Index.

Following the adoption of mobile phones, 49% of rural women actively engaged in social campaigns orchestrated by various Government Organizations (GOs) and Non-Governmental Organizations (NGOs) within their local communities. They fervently participated in campaigns centered around vaccination, tree plantation, diverse celebrations, and awareness augmentation initiatives such as those emphasizing safe drinking water, sanitation, contraception, and family planning. Their proactive involvement in these programs, pivotal for efficacious execution in rural terrains, was facilitated by the timely information and communiqués received via their mobile phones. Consequently, rural women were poised to harness their social autonomy, thereby empowering them socially. Moreover, mobile phone adoption precipitated a 43% surge in rural women's participation in group activities in the surveyed regions. Nonetheless, 47% of rural women continued to exhibit limited engagement in addressing pressing social issues like dowry and physical assault, predominantly due to societal constraints and the prevailing male-centric societal structure wherein women's perspectives are frequently sidelined.

**Fig. 3:**
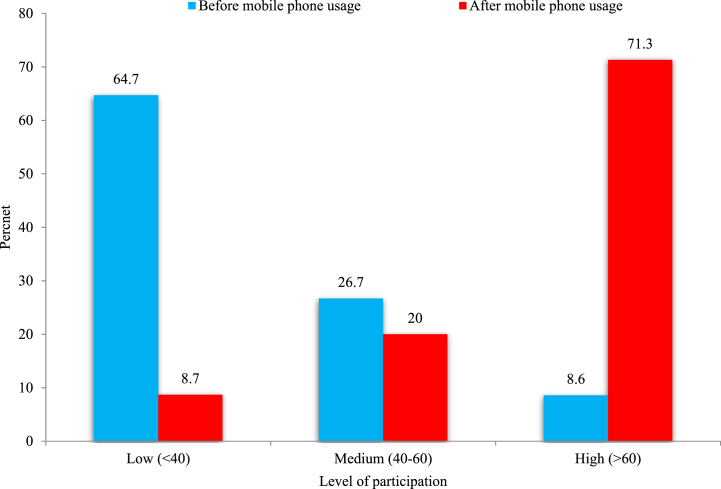
Women entrepreneur's participation to Social Activities Index (PSAI).

The Participation to Social Activities Index (PSAI) oscillated between 20% and 73% pre-mobile phone intervention and ranged from 30% to 90% post-intervention. The average PSAI escalated from 39.71% pre-mobile phone adoption to 66% post-adoption, signifying substantial strides for rural women in forging and bolstering social networks to counteract vulnerabilities inherent to social ostracization. The distribution of rural women, predicated on their PSAI value, unveils a marked paradigm shift in social participation across diverse respondent categories pre- and post-mobile phone intervention (Fig. 3).

A predominant segment, approximately two-thirds (64.7%), of the respondents exhibited limited engagement pre-mobile phone adoption. In contrast, post-adoption, an overwhelming 91.3% of rural women engaged at moderate to extensive levels in social activities. The adoption of mobile phones engendered heightened social cohesion, ameliorating feelings of isolation, amplifying their voice against societal maladies, enhancing their social prestige, standards, dignity, and fostering robust social relationships. These results are congruent with the findings of Laizu et al. [43].

#### 3.1.3 Technological empowerment

This study encompassed an examination of ten distinct technological facets. The data suggests that, prior to mobile phone adoption, a significant proportion of rural women entrepreneurs faced technological constraints. Specifically, 21% of respondents lacked any technological access, 71% had minimal access, and a mere 8% enjoyed moderate access to various technological activities. Notably, high technological access was absent among the rural women before the introduction of mobile phones. However, post-adoption, there was a discernible enhancement in rural women's technological accessibility and proficiency. Approximately 57% of rural women achieved moderate to high technological access, while 38% maintained limited access. A mere 4% remained technologically disconnected even after mobile phone adoption. On average, 42% of rural women persisted with limited or no technological access, indicating a continued empowerment deficit (Table 3).

**Table 3:** Women entrepreneur's extent of access to technological issues.

Sl. No.	Technological issues	Level of access (%)							
		Before mobile usage				After mobile usage			
		No	Low	Med.	High	No	Low	Med.	High
1.	Access to government digital services such as eksheba, 3331 KrishokBondhu Phone Seba, Krishi Call Centre 1090	6	83	11	0	0	13	68	19
2.	Knowledge and understanding about mobile financial services (MFS)	19	73	8	0	0	16	61	23
3.	Ownership and use of mobile phone for accessing digital content and platform	23	67	10	0	0	29	56	15
4.	Access and use of social media for promoting agricultural produces	24	69	7	0	0	33	48	19
5.	Understanding and use of weather forecasting	23	57	20	0	0	18	59	23
6.	Learning skill and attending training on internet for enterprise purpose	26	73	1	0	3	39	51	8
7.	Pests and diseases outbreak warning and tracking	20	66	14	0	2	68	12	8
8.	Access to modern agricultural technologies	16	82	2	0	9	52	37	2
9.	Understanding about online platform to get expert opinion	33	65	2	0	21	51	26	2
10.	Confidence to speak and access to gender-sensitive legal services such as citizen charter like dialing 109	20	77	3	0	7	61	27	4
	Average	21	71	8	0	4	38	45	12
	Mean of ATAI	28.95				55.37			
	Range of ATAI	16.7–46.7				30.0–90.0			

Scale value: No access = 0, Low access = 1, Medium access = 2 High access = 3.

ATAI = Access to Technological Activities Index.

Table 3 illustrates that, among the ten technological facets analyzed, the majority of women accessed government digital services, acquired knowledge about electronic banking, and developed an understanding of technology ownership and its utility for digital content access. They also adeptly utilized social media for agricultural product promotion and accessed weather forecasting services. The prominence of rural women's access to government digital services, facilitated by mobile phones, is particularly noteworthy [44]. Women augmented their technological acumen, skills, and awareness of digital agricultural and health services [45]. The government proffers a plethora of services via digital platforms such as eksheba, 3331 Krishok Bondhu Phone Seba, Krishi Call Centre 1090, while NGOs offer interactive voice response and live call center services.

**Fig. 4:**
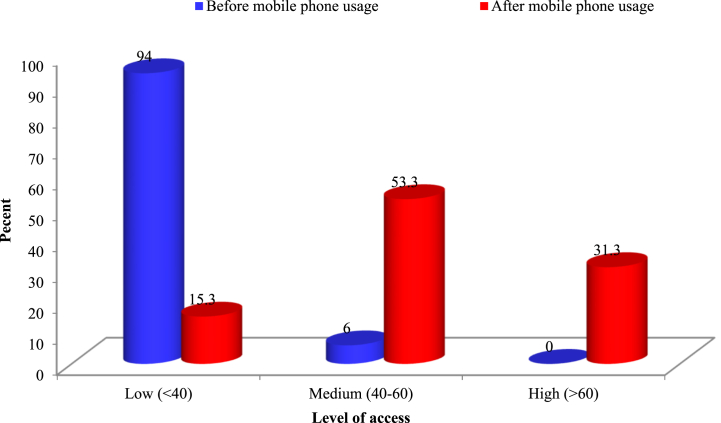
Women entrepreneur's Access to Technological Activities Index (ATAI).

The Access to Technological Activities Index (ATAI) ranged from 16.7% to 46.7% pre-mobile phone intervention, escalating to between 30% and 90% post-intervention. The mean ATAI post-mobile phone adoption was significantly elevated at 55.37%, compared to 28.95% prior, signifying a marked enhancement in rural women entrepreneurs' technological accessibility. However, the distribution of these entrepreneurs, based on their ATAI value (Fig. 4), underscores the profound progress in technological access post-mobile phone adoption.

Fig. 4 illustrates that, prior to mobile phone adoption, a staggering 94% of rural women had limited technological access. Post-adoption, a substantial 84.6% achieved moderate to high access to various technological facets. This technological empowerment facilitated their ability to operate mobile financial services, access weather-related information, and utilize social media platforms for farm product promotion, thereby elevating their economic standing. The infusion of mobile phones transformed the rural landscape, bridging the chasm between rural women and services offered by GOs and NGOs. This technological empowerment, fostering personal freedom, identity, and self-learning, resonates with the findings of Chattu et al. [32].

#### 3.1.4 Psychological empowerment

The data indicates that, prior to mobile phone adoption, rural women entrepreneurs experienced diminished psychological empowerment across various domains. Specifically, over three-quarters (77%) of respondents felt minimally empowered or not at all, 21% felt moderately empowered, and a mere 2.0% experienced high empowerment. However, post-adoption, 71.0% of respondents reported moderate to high psychological empowerment, though 29% still felt limited improvement. Notably, 70% of rural women perceived that mobile phone usage significantly bolstered their psychological well-being (Table 4). This metric is intrinsically gauged by individuals' perceptions of their quality of life experiences. Mobile phones reshaped perceptions of livelihood improvement, bolstering both physical and mental resilience.

**Table 4:** Women entrepreneur's according to their improvement in psychological issues.

Sl. #	Psychological well-being	Ability to act upon psychological well-being (%)							
		Before mobile usage				After mobile usage			
		No	Low	Med.	High	No	Low	Med.	High
1.	Articulateness and confidence in speaking with authority	7	52	39	2	0	9	45	47
2.	Attitude on own self-esteem	5	63	32	0	0	7	61	32
3.	Feeling more valued and respected	1	58	39	2	0	11	39	49
4.	Feeling of enthusiastic	8	67	17	8	0	13	51	36
5.	Feeling of self-reliance	7	75	13	4	0	14	59	27
6.	Feeling of family security	21	46	33	0	1	7	47	45
7.	Talk with unknown person	1	85	14	0	0	14	35	51
8.	Feeling of belongingness	5	79	10	6	0	49	33	18
9.	Freedom to do thing	3	85	12	0	3	2	37	59
10.	Self-wellbeing and happiness	7	88	5	0	1	13	15	70
	Average	7	70	21	2	0	29	42	29
	Mean of PWI	39.78				66.15			
	Range of PWI	26.7–70.0				30.0–86.7			

Scale value: No change = 0, Low change = 1, Medium change = 2 and High change = 3.

PWI = Psychological Well-being Index.

The Psychological Well-being Index (PWI) spanned from 26.7% to 70% pre-mobile phone adoption and ranged from 30% to 86.7% post-adoption. The average PWI witnessed a significant surge from 39.78% to 66.15%, indicating a marked improvement in rural women's psychological empowerment. However, the distribution of these women, based on their PWI, reveals disparities in psychological well-being improvement pre- and post-mobile phone adoption.

#### 3.1.5 Political empowerment

Data suggests that, prior to mobile phone adoption, rural women's engagement in political matters was minimal. A vast majority (85%) exhibited limited to no political engagement, 14% had moderate engagement, and a mere 1% displayed high engagement in various political matters. However, post-adoption, there was a significant surge in political engagement, with almost two-thirds (64%) of rural women entrepreneurs exhibiting moderate to high engagement, while just over one-third (34%) maintained limited engagement. A negligible 2% remained disengaged even post-mobile phone adoption (Table 5).

**Table 5:** Women entrepreneur's extent of participation in political issues.

Sl. #	Political issues	Level of participation (%)							
		Before mobile usage				After mobile usage			
		No	Low	Med.	High	No	Low	Med.	High
1	Participation in local policy dialogue	0	71	27	2	0	8	51	41
2	Contact with political-administrative authorities	1	76	23	0	0	19	63	19
3	Protesting against high-handedness of government officials	9	74	15	2	0	28	45	27
4	Networking and lobbying (*tadbir*) with political authorities	17	66	15	2	3	21	46	30
5	Participation in casting vote in all elections	11	85	2	2	4	29	41	26
6	Complaining against child and women violence	7	89	4	0	5	15	65	15
7	Awareness about drug addiction among the younger generation	5	83	11	0	0	58	34	8
8	Protesting against misappropriation of relief goods	2	84	14	0	5	41	44	10
9	Raising voice to ensure fair price of agricultural produce	8	75	17	0	2	57	35	6
10	Campaign for a political candidate	7	78	15	0	2	63	17	18
	Average	7	78	14	1	2	34	44	20
	Mean of PPAI	36.42				60.66			
	Range of PPAI	20.0–70.0				33.3–83.3			

Scale value: No participation = 0, Low participation = 1, Medium participation = 2 and High participation = 3; PPAI = Participation to Political Activities Index.

**Fig. 5:**
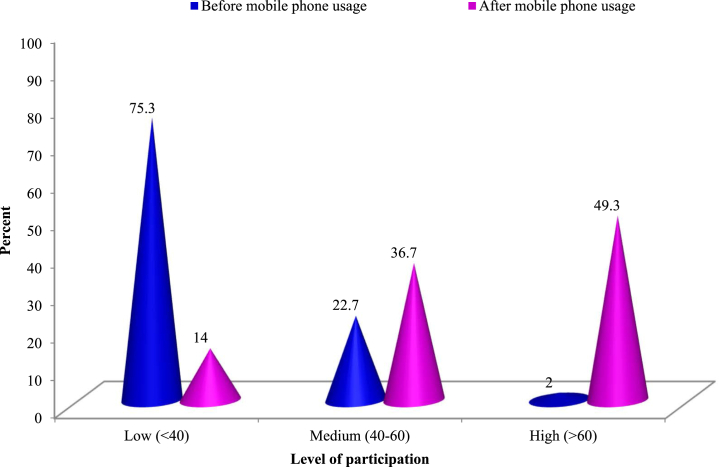
Political participation index (PPI) of women entrepreneurs.

The range of the Participation to Political Activities Index (PPAI) before and after mobile phone usage was 20%–70% and 33.3%–83.3%, respectively. The average PPAI experienced a significant improvement, from 36.42% before mobile phone usage to 60.66% after, representing a remarkable enhancement in rural women's political empowerment. The distribution of rural women, based on their PPAI, reveals substantial variations among respondents regarding their participation in political activities before and after mobile phone usage (Fig. 5).

Fig. 5 illustrates that, prior to mobile phone adoption, a significant 75.3% of respondents exhibited limited political participation, 22.7% demonstrated moderate participation, and a mere 2% were highly engaged. Subsequent to mobile phone adoption, political participation witnessed a marked enhancement, with a majority (86%) achieving moderate to high participation levels, while only 14% continued to exhibit limited participation. This augmentation can be attributed to heightened awareness, voice, income confidence, and increased participation in social and developmental activities facilitated by mobile phone usage. Moreover, the community exposure level of rural farm women amplified post-mobile phone adoption, bolstering their political empowerment and engagement in various political matters. These observations are consistent with the findings of Pahuja [46], who discerned that a majority of mobile phone users (60.67%) possessed a medium level of political empowerment, followed by high (21.33%) and low (18%) levels, respectively.

### 3.2 Improvement in empowerment dimensions after usage of mobile phone

Rural women entrepreneurs manifested significant enhancements across all empowerment dimensions subsequent to mobile phone adoption (Table 6). Every dimension, encompassing economic, social, technological, psychological, and political facets, registered marked progress post-mobile phone integration into their farm enterprises.

**Table 6:** Changes in the value of empowerment dimensions as a result of mobile phone usage.

Empowerment Dimensions	Mean Empowerment Index (MEI) values		Change in MEI	Correlation coefficient	t-value	Sig. level
	Before	After				
1. Economic empowerment	33.28	61.42	28.14	0.668	31.12	0.000
2. Social empowerment	39.71	66.00	26.29	0.574	26.38	0.000
3. Technological empowerment	28.95	55.37	26.42	0.696	30.73	0.000
4. Psychological empowerment	39.78	66.16	26.38	0.637	31.67	0.000
5. Political empowerment	36.41	60.66	24.25	0.623	25.34	0.000
Composite empowerment	37.47	61.92	24.45	0.665	33.53	0.000

The data suggests that all five empowerment dimensions underwent positive, statistically significant transformations. The most pronounced advancement was discerned in economic empowerment (28.14%), while the most modest progression was observed in political empowerment (24.25%). The other three dimensions—social (26.29%), technological (26.42%), and psychological (26.38%)—exhibited comparable shifts, yet these alterations were significant both pre- and post-mobile phone adoption.

**Fig. 6:**
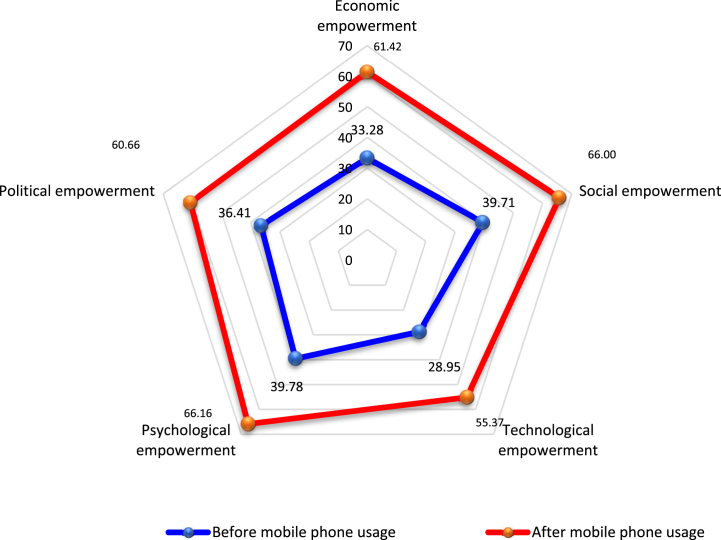
Spider diagram of before and after mean empowerment index value.

The spider diagram (Fig. 6) delineates that the post-adoption means empowerment index value peaked in the psychological dimension (66.16%) and the social dimension (66.00%), succeeded by the economic dimension (61.42%), political dimension (60.66%), and technological dimension (55.37%), in that order. This suggests that mobile phones primarily bolstered psychological fortitude, closely trailed by social empowerment. Enhanced psychological resilience empowers rural women to cultivate confidence, facilitating superior economic outcomes. The preeminent psychological mean index value signifies a paradigm shift in rural women's perceptions of mobile phones [47], while the social empowerment index value infers that mobile phone bestow social prestige upon rural women, fostering community development through augmented societal interactions [42].

Moreover, the mean composite empowerment index value was significantly elevated (61.92%) at a 1% level of significance post-mobile phone adoption, underscoring the profound impact of mobile phone usage on amplifying the empowerment of rural farm women.

### 3.3 Composite empowerment of rural farm women

**Fig. 7:**
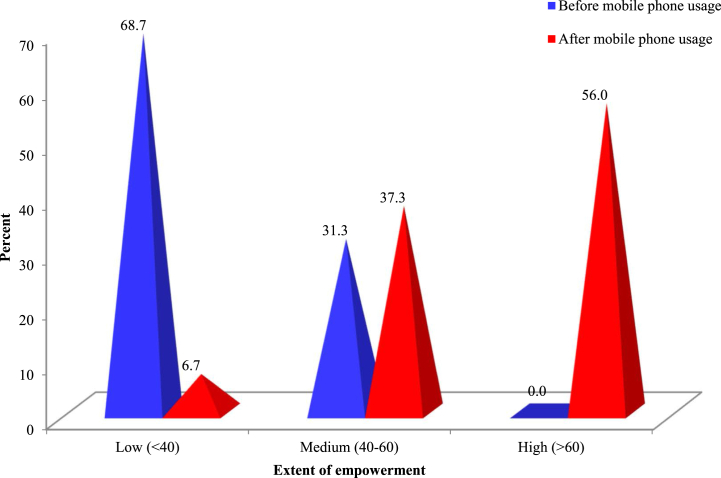
Women entrepreneur's composite empowerment index (CEI).

The composite empowerment under scrutiny encapsulates the evolution of rural farm women across economic, social, technological, psychological, and political domains post-mobile phone integration into their farm enterprises. The Composite Empowerment Index (CEI) value post-mobile phone adoption was markedly superior (61.92%) compared to its precursor (37.47%), epitomizing a transformative shift in rural women's empowerment (Fig. 7). Nonetheless, the distribution of rural farm women, predicated on their composite empowerment index, unveils a conspicuous enhancement across respondent categories concerning their empowerment levels post-mobile phone integration into their farm enterprises. Fig. 7 portrays the distribution of rural women entrepreneurs based on their Composite Empowerment Index (CEI) post-mobile phone adoption.

A remarkable 93.3% of respondents ascended to a medium to high empowerment level post mobile phone adoption. Yet, approximately 6.7% of respondents persisted at a limited empowerment level, potentially due to factors such as diminished confidence, inadequate digital proficiency, financial constraints, and societal impediments [48]. Targeted initiatives and interventions by relevant stakeholders can further amplify the empowerment trajectory of rural women via mobile phones [49]. A more nuanced understanding of mobile phone adoption and empowerment is elucidated in Table 7.

**Table 7:** Extent of usage of mobile phones and empowerment of rural farm women.

Extent of usage of mobile phone	n	Empowerment of rural farm women				Empowerment Indices	χ^2^-value
		Low (<40)	Medium (40–60)	High (>60)	Total%		
Low (up to 6)	36	44.4	47.2	8.3	100.0	163.9	50.40**
Medium (7–12)	55	7.3	56.4	36.4	100.0	229.1	
High (>12)	59	3.4	32.2	64.4	100.0	261.0	
Total	150						

**Significant at the 0.01 level of probability, df = 4.

The data delineated in Table 7 highlights discernible disparities in the empowerment levels of rural farm women, contingent upon their mobile phone usage patterns. Among those with high mobile phone usage, a significant 64.4% exhibited high empowerment, in stark contrast to 36.4% in the medium usage bracket and 8.3% in the low usage bracket. Inversely, the prevalence of low empowerment was most pronounced (44.4%) among those with minimal mobile phone usage, compared to 7.3% in the medium usage group and a mere 3.4% in the high usage group. These disparities were statistically significant at the 0.01 probability level, as evidenced by the chi-square value of 50.40. As a result, the null hypothesis was refuted, confirming a significant association between mobile phone usage by rural farm women and their empowerment [10]. The empowerment indices consistently escalated from the low usage bracket to the high usage bracket, underscoring a positive correlation. This observation corroborates the assertion that mobile phone usage markedly bolsters the empowerment of rural farm women.

## 4 Conclusion

This research probed the implications of mobile phone adoption on the empowerment of rural farm women entrepreneurs, concentrating on five pivotal empowerment dimensions: economic, social, technological, psychological, and political. The empirical evidence suggests that mobile phone adoption has engendered salutary shifts across all these dimensions, with these shifts being statistically significant. Nevertheless, economic empowerment registered the most pronounced augmentation, while political empowerment witnessed the most modest enhancement. Variations in the social, technological, and psychological dimensions appeared relatively congruent, but the post-mobile phone adoption mean empowerment index was conspicuously elevated in the psychological dimension relative to its counterparts. These findings argue that mobile phones have been instrumental in engendering significant advancements across the studied empowerment dimensions, with the overarching composite empowerment index being appreciably augmented post-mobile phone adoption. This accentuates the transformative role of mobile phones in the empowerment trajectory of rural farm women, traditionally marginalized and subjected to multifaceted disadvantages. The proliferation of mobile phone services has emerged as a catalyst, mitigating these historical imbalances. The research underscores that mobile phone adoption and its ancillary services have conferred tangible benefits upon enterprises, catalyzing the economic and psychological ascension of women entrepreneurs. It is posited that mobile phones function as pivotal instruments for engendering empowerment across diverse dimensions, underpinning activities germane to each dimension. For instance, mobile phone services and applications capacitate rural women to enhance their enterprises, fostering empowerment and thereby augmenting their holistic well-being and advancement.

However, this research, conducted in rural Bangladesh, is not devoid of limitations. The circumscribed geographical purview of this study might curtail the extrapolation of its findings to disparate socio-economic and cultural milieus. The sample's non-exhaustive representation of the entire demographic of rural women entrepreneurs in the region might circumscribe the generalizability of the insights. The reliance on self-reported data might engender biases, potentially influenced by social desirability. Moreover, the research did not delve into potential variances in mobile phone technology and usage patterns.

Future research should expand the geographical ambit and encompass a more diverse and representative cohort. Longitudinal studies could furnish more robust insights regarding the causal nexus between mobile phone adoption and empowerment. The integration of qualitative research methodologies might yield a more intricate understanding of the experiential nuances encountered by rural women entrepreneurs. Subsequent research should also probe policy implications, incorporate emergent technologies, and scrutinize effects on specific sectors to furnish a comprehensive understanding of the merits and demerits inherent in leveraging mobile phones for the empowerment of rural women entrepreneurs.

## Ethical approval

The ethical committee of Bangabandhu Sheikh Mujibur Rahman Agricultural University approved this study. Besides, all ethical guidelines were followed during the research process. This study also took a prior verbal approval of each respondent before face-to-face interview.

## Funding

This study acknowledges the funding support of the Ministry of Science and Technology, Government of the People's Republic of Bangladesh.

## Data availability

The data will be made available on request.

## CRediT authorship contribution statement

**Md Sadekur Rahman:** Writing – review & editing, Writing – original draft, Validation, Software, Methodology, Formal analysis, Data curation, Conceptualization. **Md Enamul Haque:** Writing – review & editing, Writing – original draft, Visualization, Validation, Supervision, Software, Resources, Project administration, Methodology, Investigation, Formal analysis. **Md Safiul Islam Afrad:** Writing – review & editing, Writing – original draft, Visualization, Validation, Supervision, Software, Resources, Project administration, Methodology, Investigation, Formal analysis. **Shaikh Shamim Hasan:** Writing – review & editing, Writing – original draft, Visualization, Validation, Software, Resources, Project administration, Methodology, Formal analysis, Data curation. **Md Abiar Rahman:** Writing – review & editing, Writing – original draft, Visualization, Validation, Resources, Methodology, Formal analysis, Data curation.

## Declaration of competing interest

The authors declare that they have no known competing financial interests or personal relationships that could have appeared to influence the work reported in this paper.
